# High-risk factors associated with persistence and progression of cervical intraepithelial neoplasia 2 in women of reproductive age undergoing conservative management: a retrospective analysis

**DOI:** 10.3389/fpubh.2026.1961644

**Published:** 2026-10-21

**Authors:** Meilian Chen, Gang Xiao, Hongmei Xiao, Yingxue Bai, Yixue Zhou, Xuelian Tan, Limei Wu, Haixia Jiang

**Affiliations:** 1 Department of Obstetrics and Gynecology, West China School of Medicine, Sichuan University, Sichuan University Affiliated Chengdu Second People’s Hospital, Chengdu Second People’s Hospital, Chengdu, China; 2 Department of Gastrointestinal Surgery, West China School of Medicine, Sichuan University, Sichuan University Affiliated Chengdu Second People’s Hospital, Chengdu Second People’s Hospital, Chengdu, China

**Keywords:** cervical intraepithelial neoplasia 2, conservative management, high-grade squamous intraepithelial lesion, human papillomavirus type 16/18, P16/Ki-67, persistence, progression

## Abstract

**Objective:**

To investigate the high-risk factors associated with the persistence or progression of cervical intraepithelial neoplasia 2 (CIN2) to cervical intraepithelial neoplasia 3 (CIN3) in women of childbearing age undergoing conservative management, thereby providing guidance for selecting appropriate treatment strategies for women with CIN2 who wish to preserve their fertility.

**Methods:**

Univariate and multivariate logistic regression analyses were performed using the clinical and pathological characteristics of the included patients to identify high-risk factors associated with the persistence or progression of CIN2.

**Results:**

In total, 226 patients were included in this study. Of these, 142 (62.8%) experienced lesion regression, 58 (25.7%) had persistent CIN2, and 26 (11.5%) progressed to CIN3. Univariate analysis indicated that age, high- squamous intraepithelial lesion (HSIL), human papillomavirus type 16/18 (HPV16/18) positivity, and P16/Ki-67 positivity were associated with persistence of CIN2 or progression to CIN3. Multivariate analysis revealed that age of >35 years (OR 2.732, 95% CI 1.350–5.529), HPV16/18 positivity (OR 1.506, 95% CI 1.129–17.611), HSIL (OR 4.693, 95% CI 3.554–19.211), and P16/Ki-67 positivity (OR 4.007, 95% CI 2.819–6.014) were associated with persistence or progression of CIN2.

**Conclusion:**

Age of >35 years, HPV16/18 positivity, HSIL, and P16/Ki-67 positivity are associated with persistence and progression of CIN2. Conservative management with outpatient follow-up is an appropriate treatment option for carefully selected patients without these high-risk factors.

## 1 Introduction

Cervical intraepithelial neoplasia 2 (CIN2) is a precancerous lesion of the cervix; however, studies have reported that approximately 47.0%–73.5% of patients experience spontaneous regression within 2 years (1–4). Surgical resection of the cervix is the most effective treatment for CIN2. However, in women of childbearing age, this procedure increases the risk of miscarriage, preterm birth and cervical insufficiency, thereby affecting future fertility (5). Consequently, the primary challenge in the clinical management of women of childbearing age with CIN2 is to accurately identify which women should undergo surgical treatment and which can be managed conservatively with regular follow-up.

Previous retrospective analyses have identified human papillomavirus type 16 (HPV16) positivity and high-grade squamous intraepithelial lesion (HSIL) as high-risk factors for the persistence and progression of CIN2 lesions (2, 6, 7). In a prospective study of 205 patients with CIN2, Bergqvist et al. found that lesions occupying >50% of the transformation zone and the presence of HSIL were associated with CIN2 progression, while HPV genotyping had limited predictive value for clinical outcomes (8). By contrast, another multicentre prospective study conducted in Italy found that when HPV, P16/Ki-67, and FAM19A4/miR124-2 methylation were all negative, the probability of spontaneous regression of CIN2 lesions increased (9). In another prospective study involving 291 patients with CIN2, after 24 months of observation and treatment, 73.5% of patients experienced lesion regression, 14.8% had persistent CIN2, and 11.7% progressed to cervical intraepithelial neoplasia 3 (CIN3); HPV16 and HSIL were associated with the persistence and progression of CIN2 lesions (2). The conclusions of these studies vary according to the high-risk factors included in each analysis. Consequently, relevant guidelines—including the 2019 American Society for Colposcopy and Cervical Pathology (ASCCP) risk management guidelines, the 2025 joint guidance from the British Society for Colposcopy and Cervical Pathology (BSCCP) and the European Society of Gynaecological Oncology (ESGO), and relevant Chinese guidelines—all support active surveillance for women of childbearing age with biopsy-confirmed CIN2. These guidelines recommend individualised management based on cytology, HPV genotyping, colposcopic findings and the visibility of the transformation zone, however, there are discrepancies regarding the primary determinants of conservative management for CIN2 (3).

With the trend towards younger onset of CIN2 and the current pattern of later marriage and childbearing in China, the number of patients with CIN2 who wish to preserve their fertility is increasing. Achieving a balance between cancer prevention and reproductive goals in this population requires individualised diagnostic and treatment strategies. In accordance with current Chinese guidelines for the screening and treatment of cervical precancerous lesions, this study aimed to identify high-risk factors associated with the persistence or progression of CIN2 lesions to CIN3 in women of childbearing age undergoing expectant management, without increasing healthcare costs.

## 2 Methods

### 2.1 Study design

We included 226 women with biopsy-confirmed CIN2 who were treated at Sichuan University affiliated Chengdu Second People’s Hospital between 2021 and 2024. All tissue sections were independently reviewed and re-examined by two experienced gynaecological pathologists. Only patients whose CIN2 diagnosis was confirmed by both reviewers were ultimately included. Clinical and pathological data were collected, including demographic characteristics, cervical cancer screening results, colposcopic findings, and histopathological results. This study was approved by the relevant ethics committee.

The inclusion criteria were an age of 18–40 years, a fully visible transformation zone (colposcopically classified as a Type 1 or Type 2 transformation zone), and the ability to undergo long-term follow-up, with a maximum follow-up period of 48 months or until lesion persistence or progression was observed, at which point follow-up was discontinued. Patients were followed up every 6 months during the first year. If results were negative, follow-up was conducted every 6–12 months during the second year. Follow-up was concluded if no persistent lesions or progression to CIN3 were detected. Patients referred for colposcopy following routine gynaecological examination showing grossly visible lesions or abnormal ThinPrep Cytology Test (TCT) or HPV results underwent cervical biopsy, with endocervical curettage performed where necessary. If the lesion persisted, cervical conisation was performed. If the lesion progressed, cervical conisation or other treatment was performed in accordance with cervical cancer guidelines. Following treatment, patients were withdrawn from the study follow-up.

The exclusion criteria were previous cervical conization or other treatment for CIN2, a history of other malignant tumors or sexually transmitted infections, a history of treatment for other cervical diseases, poor or compromised immune function, and pregnancy or breastfeeding. Patients with a type 3 transformation zone were excluded because the incomplete visualization of the transformation zone may increase the risk of missing occult high-grade cervical lesions, making conservative management inappropriate.

### 2.2 Definition of clinical outcomes

Regression was defined as downgrading to CIN1 confirmed by cervical biopsy, or negative results for both TCT and HPV screening during the study follow-up period. Persistence was defined as no change in the patient’s CIN2 diagnosis following histological assessment during follow-up. Progression was defined as pathologically confirmed CIN3 or cervical cancer at any point during the study.

### 2.3 HPV detection and genotyping

Fluorescent polymerase chain reaction technology was used for HPV genotyping, and all procedures were carried out strictly in accordance with the manufacturer’s instructions for the kit (HybriBio Ltd). The assay detected 17 high-risk HPV types (16, 18, 31, 33, 35, 39, 45, 51, 52, 53, 56, 58, 59, 66, 68, 73, and 82). HPV16 and HPV18 were analysed separately because of their high carcinogenic potential, whereas infection with the remaining high-risk HPV types was classified as HR-HPV (non-16/18).

### 2.4 TCT testing

For cervical TCT testing, a liquid-based cytology system [BD PrepStain™ Slide Processor, BD Medical Devices (Shanghai) Co., Ltd.] was used to prepare thin smears. Following smear preparation, the specimens were interpreted independently by two experienced gynaecological pathologists at our hospital in accordance with the Bethesda 2001 classification system (10).

### 2.5 P16/Ki-67 testing

Immunohistochemical staining for P16/Ki-67 was performed on cervical biopsy tissue specimens, with all procedures carried out strictly in accordance with the manufacturer’s instructions. P16 positivity was defined as strong and diffuse staining, and Ki-67 positivity was defined as staining in ≥50% of cells. P16/Ki-67 was considered positive when both criteria were met.

### 2.6 Statistical methods

Statistical analysis was performed using SPSS version 23.0. Continuous variables are presented as mean ± standard deviation, whereas categorical variables are presented as percentage (%). Comparisons of continuous variables were performed using the independent-samples t-test, whereas comparisons of categorical variables were performed using the chi-squared test. Univariate and multivariate logistic regression analyses were subsequently performed to identify clinical and pathological risk factors associated with the persistence or progression of CIN2 lesions. A *p* value of <0.05 was considered statistically significant. The variables included age, parity, number of deliveries, economic status, contraceptive use, HPV infection status (HPV16/18 or other high-risk HPV types), TCT findings, type of colposcopic transformation zone, extent of lesion involvement across colposcopic quadrants (1–2 or 3–4), P16/Ki-67 status, and cervical gland involvement.

## 3 Results

In total, 226 patients were included in this study. Of these, 142 (62.8%) experienced lesion regression, 58 (25.7%) had persistent CIN2, and 26 (11.5%) progressed to CIN3; no patients progressed to cervical cancer during the follow-up period. Among the included patients, 21 (9.3%) used oral contraceptives, while 141 (62.4%) did not use any contraception. Liquid-based cytology results showed negative for intraepithelial lesion or malignancy (NILM) in 26 (11.5%) cases, atypical squamous cells of undetermined significance (ASC-US) in 65 (28.8%) cases, atypical squamous cells—cannot exclude HSIL (ASC-H) in 25 (11.1%) cases, low-grade squamous intraepithelial lesion (LSIL) in 22 (9.7%) cases, and HSIL in 88 (38.9%) cases. Among all patients, 151 (66.8%) were HPV16/18-positive. Pathological examination of cervical biopsy specimens revealed P16/Ki-67 double-positive staining in 57 (25.2%) cases, while 169 (74.8%) cases were negative. In 206 (91.2%) cases, the lesions did not involve the cervical glands, whereas biopsies from 20 (8.8%) patients demonstrated CIN2 with glandular involvement. Colposcopic examination revealed that 189 (83.6%) patients had a Type 1 transformation zone. The lesion involved 1–2 quadrants in 190 (84.1%) patients and 3–4 quadrants in 36 (15.9%) patients. The relevant clinical and pathological data are presented in Table 1.

**Table 1:** Clinical features of 226 patients.

Features		Number of patients (*n*)	Percentage (%)
Age (y), mean±SD		30.75 ± 4.240	
Gravity (*n*)	0	10	4.4
	1	28	12.4
	2	64	28.3
	3	51	22.6
	4	40	17.7
	≥5	33	14.6
Parity (*n*)	0	15	6.6
	1	94	41.6
	2	102	45.1
	3	15	6.7
Contraception	None	141	62.4
	COC	21	9.3
	Condom	18	8.0
	Intrauterine device	46	20.4
Economic conditions	Rural	142	62.8
	Urban	84	37.2
Cytology	NILM	26	11.5
	ASCUS	65	28.8
	ASC-H	25	11.1
	LSIL	22	9.7
	HSIL	88	38.9
HPV genotyping	Negative	5	2.2
	16/18	151	66.8
	HR HPV (non-16/18)	70	30.1
P16/ki67 dual stain	Negative	169	74.8
	Positive	57	25.2
Gland involvement	Negative	206	91.2
	Positive	20	8.8
Type of TZ	TZ1	189	83.6
	TZ2	37	16.4
Lesion size	1–2 quadrants	190	84.1
	3–4 quadrants	36	15.9
Clinical outcomes	Regression	142	62.8
	Persistence	58	25.7
	Progression	26	11.5

HPV, Human papillomavirus; TZ, Transformation zone; COC, Combined oral contraceptive; ASCUS, Atypical squamous cells of undetermined significance; ASC-H, Atypical squamous cell cannot exclude high-grade squamous intraepithelial lesion; LSIL, Low-grade squamous intraepithelial lesion; HSIL, High-grade squamous intraepithelial lesion; NILM, Intraepithelial lesions or malignancy; HR HPV, high-risk human papillomavirus; TZ, transformation zone; SD, standard deviation.

We further investigated the high-risk factors associated with the persistence or progression of CIN2 lesions. Univariate analysis indicated that age, HSIL, HPV16/18 positivity and P16/Ki-67 positivity were associated with the persistence of CIN2 lesions or progression to CIN3 (*p* < 0.05) (Table 2). Multivariate analysis revealed that age of >35 years (Odds ratio [OR] 2.732, 95% confidence interval [CI] 1.350–5.529; *p* = 0.012), HPV16/18 positivity (OR 1.506, 95% CI 1.129–17.611; *p* = 0.004), P16/Ki-67 positivity (OR 4.007, 95% CI 2.819–6.014; *p* = 0.001), and HSIL (OR 4.693, 95% CI 3.554–19.211; *p* = 0.019) were associated with the persistence or progression of CIN2 lesions. However, transformation zone type, extent of cervical involvement, oral contraceptive use, number of pregnancies, number of deliveries, economic status and cervical gland involvement were not associated with spontaneous regression of CIN2 lesions.

**Table 2:** Risk differences and risk ratios for regression and persistence/progression of characteristics and baseline findings among 226 women.

Factors	Univariate analysis			Multivariate analysis		
	Regression (n, %)	Persistence and progression (n, %)	*p*-value	Odds ratio	95% CI	*p*-value
Patients	142	84				
Age			0.010			0.012
≥18y, ≤30y	83(58.5)	32(38.1)		Ref.		
>30y, ≤35y	36(25.4)	35(41.7)		2.247	0.980–5.151	0.056
>35y, ≤40y	23(16.2)	17(20.2)		2.732	1.350–5.529	0.005
Gravity (*n*)			0.233			
0	4(2.8)	6(7.1)				
1	18(12.7)	10(11.9)				
2	35(24.6)	29(34.5)				
3	34(23.9)	17(20.2)				
4	26(18.3)	14(16.7)				
≥5	25(17.6)	8(9.5)				
Parity (*n*)			0.256			
0	8(5.6)	7(8.3)				
1	65(45.8)	29(34.5)				
2	61(43.0)	41(48.8)				
3	6(4.2)	7(8.3)				
4	2(1.4)	0(0.0)				
Contraception			0.733			
None	85(59.9)	56(66.7)				
COC	15(10.6)	6(7.1)				
Condom	12(8.5)	6(7.1)				
Intrauterine device	30(21.1)	16(19.0)				
Economic conditions			0.156			
Rural	84(59.2)	58(69.0)				
Urban	58(40.8)	26(31.0)				
Cytology			0.003			0.019
NILM	25(17.6)	1(1.2)		Ref.		
ASCUS	40(28.2)	25(29.8)		3.345	2.102–13.110	0.008
ASC-H	15(10.6)	10(11.9)		3.103	2.157–17.053	0.001
LSIL	10(7.0)	12(14.3)		4.272	2.399–12.275	0.003
HSIL	52(36.6)	36(42.9)		4.693	3.554–19.211	0.006
HPV genotyping			0.002			0.004
Negative	4(2.8)	1(1.1)		Ref.		
HPV 16/18	83(58.5)	68(81.0)		1.506	1.129–17.611	0.036
HR HPV (non-16/18)	55(38.7)	15(17.9)		1.014	0.056–5.207	0.494
P16/Ki67 dual stain			0.039			0.001
Negative	113(79.6)	56(66.7)		Ref.		
Positive	29(20.4)	28(33.3)		4.007	2.819–6.014	
Gland involvement			0.448			
Negative	131(92.3)	75(89.3)				
Positive	11(7.7)	9(10.7)				
Type of TZ			0.195			
TZ1	115(81.0)	74(88.1)				
TZ2	27(19.0)	10(11.9)				
Lesion size			0.852			
1–2 quadrants	120(84.5)	70(83.3)				
3–4 quadrants	22(15.5)	14(16.7)				

HPV, Human papillomavirus; TZ, Transformation zone; COC, Combined oral contraceptive; ASCUS, Atypical squamous cells of undetermined significance; ASC-H, Atypical squamous cell cannot exclude high-grade squamous intraepithelial lesion; LSIL, Low-grade squamous intraepithelial lesion; HSIL, High-grade squamous intraepithelial lesion; NILM, Intraepithelial lesions or malignancy; HR HPV, high-risk human papillomavirus; TZ, transformation zone; SD, standard deviation; 95% CI, 95% Confidence interval.

## 4 Discussion

Previous studies have identified age, smoking, HPV infection, immune dysfunction, and HSIL as high-risk factors influencing the persistence and progression of CIN2 lesions (2, 7–9). In the present study, univariate and multivariate analyses indicated that age of >35 years, HPV16/18 positivity, HSIL, and P16/Ki-67 positivity were high-risk factors for the persistence and progression of CIN2.

Previous studies have found that women under 30 years of age with CIN2 have a higher rate of spontaneous regression. Other studies have also reported that women aged 25 years or younger with CIN2 have an extremely low risk of progression to cervical cancer and are therefore better suited to conservative management (11, 12). While numerous studies have focused on women under 30 years of age, between 2011 and 2019 the number of pregnant women aged >35 years in China increased by 75%, this change underscores the need to place greater emphasis on conservative management for women aged ≥35 years with CIN2 (13). According to the National Audit of the British Society for Colposcopy and Cervical Pathology, conservative management of CIN2 in women aged ≥40 years is a relative contraindication. Furthermore, because fertility declines significantly after the age of 40 years, this study included women aged ≤40 years (14). The study identified high-risk factors associated with the persistence and progression of CIN2 in women aged >35 years. These findings are consistent with those of Wang et al. (15, 16). Previous studies have shown that patients aged 25–40 years with biopsy-confirmed CIN2 have a significantly higher risk of being diagnosed with CIN3 on postoperative histopathological examination following a loop electrosurgical excision procedure than patients aged ≤25 years. It has therefore been hypothesised that age is positively correlated with the risk of residual CIN3 following cervical biopsy. Consequently, women aged >35 years with CIN2 have a higher risk of persistent lesions and progression to CIN3. Because the vast majority of women in this study were aged ≤35 years, conservative management of CIN2 is likely to benefit a greater proportion of patients.

This study found that HPV16/18 positivity, HSIL, and P16/Ki-67 positivity were associated with the persistence of CIN2 lesions and progression to CIN3. Persistent infection with high-risk HPV is a major cause of CIN and cervical cancer, particularly HPV16/18. HPV types 16 and 18 possess multiple immune evasion mechanisms that make them difficult for the host immune system to clear, leading to persistent infection. Following infection, the viral genome can continuously express E6/E7 proteins in cervical epithelial cells, inducing malignant transformation and thereby contributing to the persistence of CIN2 lesions or progression to CIN3, and ultimately cervical cancer. HSIL typically occurs when cellular atypia is most pronounced and proliferative activity is greatest. At this stage, the normal differentiation programme of cervical epithelial cells has been severely disrupted and cell proliferation has become uncontrolled, resulting in a low rate of spontaneous clearance of CIN2 lesions. Notably, this HPV-driven mechanism is not restricted to the cervix, and CIN, VaIN and vulvar intraepithelial neoplasia are increasingly viewed as site-specific manifestations of a single “field disease” with a common aetiological agent and largely overlapping risk factors (17). Consistent with the present findings, previous studies of VaIN have reported an association with high-risk HPV and have shown that conservative management with watchful waiting may be appropriate for a subset of patients (18, 19). By analogy, some patients with CIN2 may also be managed conservatively with regular follow-up. P16/Ki-67 double-positive staining is associated with disrupted cell-cycle regulation and abnormal proliferation. P16 over expression is a key marker of cell-cycle dysregulation, whereas high Ki-67 expression directly reflects abnormal cell proliferation. P16/Ki-67 double-positive staining therefore indicates both severe disruption of cell-cycle regulation and abnormally active cell proliferation and is consequently associated with the persistence of CIN2 lesions and progression to CIN3.

The strength of this study lies in its integration with China’s current cervical cancer screening strategy. Against the backdrop of the country’s evolving three-child policy and demographic trends towards later marriage and childbearing, the study suggests that regular follow-up is a viable treatment option for women of childbearing age with biopsy-confirmed CIN2. The high-risk factors included in the study are consistent with current Chinese cervical cancer screening guidelines and are applicable in the vast majority of hospitals throughout the country. All tissue samples were examined and independently reviewed by pathologists specialising in gynaecological pathology, while colposcopy was performed by senior colposcopy specialists, lending credibility to the study findings. The principal limitation of this study is its retrospective design, which introduces the potential for selection and information bias. Because this was a single-centre study, its generalisability may be limited. Another limitation is the lack of information regarding HPV vaccination status. HPV vaccination may influence HPV persistence and the natural regression of cervical intraepithelial lesions, and vaccination coverage may differ according to age (20). Therefore, the relatively higher regression rate observed among younger women in our cohort may have been partly influenced by differences in HPV vaccination status, which could not be assessed because these data were not systematically available. Future prospective studies should incorporate HPV vaccination history together with HPV genotype, smoking status, and other relevant behavioral and clinical factors to more accurately evaluate the natural history of CIN2.

In summary, the spontaneous regression rate of CIN2 is high. Individualised management can be implemented by considering the patient’s age, HPV genotype, TCT results, and histopathological findings. Carefully selected women do not appear to have an increased risk of progression to CIN3. Conservative management with regular follow-up is recommended for patients aged ≤35 years who are HPV16/18-negative, are P16/Ki-67-negative, have TCT results showing no HSIL or higher-grade lesions, and wish to preserve their fertility.

## Data Availability

The raw data supporting the conclusions of this article will be made available by the authors, without undue reservation.
